# Managing Complex Chronic Otitis Media: Insights from Subtotal Petrosectomy with Blind Sac Closure

**DOI:** 10.3390/jcm14248633

**Published:** 2025-12-05

**Authors:** Angelo Immordino, Simone Oliva, Palmira Immordino, Federico Sireci, Francesco Lorusso, Riccardo Manzella, Salvatore Gallina, Antonino Maniaci, Giannicola Iannella, Quentin Mat, Francesco Dispenza

**Affiliations:** 1 Otorhinolaringology Section, Department of Biomedicine, Neuroscience and Advanced Diagnostics, University of Palermo, 90127 Palermo, Italy; simone.oliva@community.unipa.it (S.O.); francesco.lorusso@policlinico.pa.it (F.L.); riccardo.manzella@community.unipa.it (R.M.); salvatore.gallina@unipa.it (S.G.); francesco.dispenza@unipa.it (F.D.); 2 Hygiene and Preventive Medicine Section, Department of Health Promotion, Maternal and Infant Care, Internal Medicine and Medical Specialties, University of Palermo, 90127 Palermo, Italy; palmira.immordino@unipa.it; 3 Otorhinolaryngology Section, Department of Precision Medicine in Medical, Surgical and Critical Care (Me.Pre.C.C.), University of Palermo, 90127 Palermo, Italy; federico.sireci@unipa.it; 4 Faculty of Medicine and Surgery, University of Enna “Kore”, 94100 Enna, Italy; antonino.maniaci@unikore.it; 5 Department of Organi di Senso, Sapienza University, 00185 Rome, Italy; giannicola.iannella@uniroma1.it; 6 Department of Otorhinolaryngology, Centre Hospitalier Universitaire Charleroi, 6000 Charleroi, Belgium; quentin.mat@chu-charleroi.be

**Keywords:** chronic otitis media, chronic middle ear disease, middle ear surgery, subtotal petrosectomy, middle ear obliteration

## Abstract

**Objectives**: Chronic otitis media poses a surgical challenge, and subtotal petrosectomy represents a last-resort intervention. This study aims to evaluate the efficacy and safety of subtotal petrosectomy with blind sac closure of the external auditory canal in managing chronic and recurrent otitis media by sharing our experiences and discussing the findings from a comprehensive literature review. **Methods**: A retrospective analysis including nine patients undergoing subtotal petrosectomy with blind sac closure of the external auditory canal was conducted. Additionally, two independent otolaryngologists conducted a review according to the Preferred Reporting Items for Systematic Reviews and Meta-analyses (PRISMA) statement. The criteria for considering studies for the review were based on the population, intervention, comparison, outcome, timing, and setting (PICOTS) framework. **Results**: Our retrospective analysis comprised eight chronic cholesteatomatous otitis media cases and one atelectatic case. No postoperative complications occurred. The literature review discussed SP applications, surgical techniques, and outcomes from 17 selected studies. **Conclusions**: Subtotal petrosectomy with blind sac closure of the external auditory canal effectively manages chronic otitis, exhibiting minimal complications and improved postoperative outcomes. Despite study limitations, including a small sample size, this research provides valuable insights into subtotal petrosectomy’s application and success. The literature review enhances understanding by summarizing findings from diverse studies, offering a comprehensive view of the procedure’s evolution and applications.

## 1. Introduction

The primary therapeutic objectives in the surgical treatment of chronic otitis media are to clear out the disease and, when feasible, to reconstruct the patient’s hearing. Subtotal petrosectomy (SP) represents a surgical intervention frequently employed as a last resort in addressing chronic and recurrent middle ear disorders, with or without cholesteatoma, in individuals experiencing significant conductive hearing loss that is not amenable to surgical correction [1,2]. In 1958, Thomas Rambo pioneered a radical technique for this procedure, which involved performing a canal wall down mastoidectomy, closing the eustachian tube (ET), obliterating the tympano-mastoid cavity with adipose tissue, and implementing a blind sac closure (BSC) of the external auditory canal (EAC). He recommended this option for patients with compromised hearing or those with limited prospects for hearing restoration [3]. Subsequently, the procedure has been described in the literature using various terms, including middle ear obliteration, total ear canal elimination, and subtotal petrosectomy. Over the years, both the surgical technique and its indications have evolved. The modern concept of SP, introduced by Ugo Fisch in 1986, is now widely accepted among otologists [4]. The prevailing theme in the literature emphasizes establishing a secure ear with minimized risks of discharge and disease recurrence, enhancing postoperative care, and improving patients’ quality of life. In recent decades, the indications for SP have expanded to include the treatment of osteoradionecrosis, large tumors in the tympano-mastoid region (e.g., paragangliomas B2/3 and facial nerve tumors, squamous cell carcinoma of the EAC), temporal bone fractures involving the otic capsule, meningoencephalic herniation, iatrogenic cerebrospinal fluid leakage, inner ear malformations, complex cases of cochlear implant (CI) surgery, and other implantable devices [1,5]. This study aims to evaluate the efficacy and safety of SP with BSC of the EAC in managing chronic and recurrent otitis media. We also seek to identify appropriate indications for this technique by sharing our experiences and discussing the findings from a comprehensive literature review.

## 2. Materials and Methods

### 2.1. Study Sample

We conducted a retrospective analysis of patients affected by recurrent chronic middle ear disease who underwent SP with BSC of the EAC from 2016 to 2024 at the ENT Department of our University Hospital. The study was conducted in accordance with the Declaration of Helsinki and approved by the Institutional Review Board of Policlinico “Paolo Giaccone” of Palermo (11–22 April 2024). Each participant underwent a comprehensive anamnesis, gathering personal information, symptoms onset, hearing complaints, and a review of any previous medical and/or surgical treatments. Additionally, a thorough otolaryngologic examination was performed, including micro-otoscopy and endoscopy of the upper airways. Audiometric testing assessed the pure-tone average (PTA) at frequencies of 0.5, 1, 2, and 4 kHz for all subjects. Our study included patients of any gender and age suffering from chronic cholesteatomatous or non-cholesteatomatous otitis media who underwent SP with BSC of the EAC, with or without CI placement. Patients undergoing the same procedure for conditions other than chronic middle ear disease were excluded from the study group. All subjects enrolled in the study underwent preoperative temporal bone computed tomography (CT). Patients indicated for cochlear implant placement also received contrasted magnetic resonance imaging (MRI) of the inner ear.

Based on the anamnestic, clinical, and radiological data, patients were informed about possible surgical strategies.

Patient selection for SP with BSC followed predefined clinical and radiological criteria. The procedure was indicated in cases of recurrent chronic middle ear disease in which standard tympanomastoid techniques were unlikely to ensure long-term disease control. SP with BSC was routinely chosen for patients with a history of previous ear surgery, for individuals in whom reliable postoperative follow-up could not be guaranteed (such as patients with autism spectrum disorder or those unable to attend regular evaluations), and for patients initially scheduled for canal-wall–down mastoidectomy but in whom intraoperative findings required a more extensive approach. The procedure was also indicated in cochlear implant candidates with chronic otologic pathology associated with a high risk of electrode extrusion. Overall, the decision-making process was based on disease extension, anticipated difficulty in achieving a safe and dry ear, and patient-related factors limiting the applicability of less demolitive techniques.

After comprehensive counseling, patients provided written consent, following the study protocol presentation. The surgical procedures were uniformly performed under general anesthesia by the same expert surgeon, using a postauricular incision to access the middle ear. The bony-cartilaginous junction of the EAC was transected, followed by the dissection and eversion of the EAC skin from the EAC cartilage. The EAC closure as a blind sac was achieved with absorbable sutures, preserving the skin flap integrity and reinforcing the closure with an additional soft tissue layer, if necessary. A secondary closure of the EAC involved suturing the mastoid periosteum to the EAC cartilage. A radical mastoidectomy was performed to remove all disease, mastoid air cells, mucosa, and structures, including the skin, tympanic membrane, malleus, and incus. For cholesteatoma matrix adherent to the dura, bipolar devitalization was preferred. Meningoencephalic herniation was managed with bipolar diathermy and reinforced with a muscle plug. Canaloplasty was performed to ensure complete removal of squamous epithelium, and the Fallopian canal (FC) was lowered for optimal cochlea and round window niche exposure. For cochlear implant candidates, a bony bed and tie-down were prepared in the skull for the receiver-stimulator, and the electrode was inserted through the round window or the cochleostomy. The ET opening was sealed with bone wax and cartilage, and the mastoid cavity was filled with a rifampicin-treated abdominal fat graft. The subcutaneous tissue and skin were closed using a two-layer technique. In patients indicated for auditory rehabilitation with a BAHA, a titanium implant and abutment were placed in the temporal bone and topped with a “healing cap” to protect the surgical wound during its healing process. A compressive dressing was applied post-surgery, and a broad-spectrum prophylactic antibiotic therapy was started. Patients were discharged 48 h post-procedure. During the first postoperative month, patients had weekly follow-up appointments for wound care and to assess surgical wound healing. After the first month, follow-up visits were scheduled once every two months for the first six months, transitioning to long-term evaluations after that. The primary short-term outcome was the healing of the surgical site. Minor complications were considered those manageable with home and/or outpatient therapy, while major complications were those requiring hospitalization and/or surgical reintervention: surgical site complications (postoperative infection of the cavity, infection, granulation, dehiscence, or fistulization of the surgical wound, development of seroma, donor site complications), presence of residual disease, sensorineural hearing loss and/or vertigo onset, and/or postoperative facial nerve paralysis. For long-term follow-up, the key outcome was the absence of pathology recurrence. Given the EAC closure, radiologic monitoring was necessary. We recommended an MRI including the tympanomastoid and petrous regions, using fat-suppressed T2 and diffusion-weighted imaging (DWI) sequences without contrast at one year post-surgery, with subsequent scans planned at three, five, and ten years for comprehensive evaluation. In patients in whom a CI was placed, MRI was performed using a 1.5 Tesla, and in selected cases with removable magnets or manufacturer-approved sequences, a 3 Tesla MRI was used. When necessary, metal-artifact reduction techniques such as Slice Encoded Metal Artifact Correction (SEMAC) or Multi-Acquisition Variable-Resonance Image Combination (MAVRIC) were applied to minimize artifacts from the implant [6].

### 2.2. Literature Review

The literature review was conducted following the Preferred Reporting Items for Systematic Reviews and Meta-analyses group (PRISMA) guidelines [7].

Two independent authors conducted a comprehensive search by consulting the main scientific databases on the web, including PubMed, Google Scholar, Medline, EMBASE, Web of Science, and the Cochrane Library. Specific keyword pairs such as “chronic otitis media” (OR “middle ear disease” OR “cholesteatoma”) AND “subtotal petrosectomy” (OR “middle ear obliteration” OR “blind sac closure”) were used for the search. Titles and abstracts were reviewed to screen out non-relevant articles, and the working group reviewed the full text of the remaining articles. The results of the studies were then combined, integrated, and analyzed.

The criteria for considering studies for the review were based on the population, intervention, comparison, outcome, timing, and settings (PICOTS) framework:

- *Population and inclusion criteria:* Studies in the English language on patients of all ages, genders, and ethnicities with chronic or recurrent middle ear disease, with or without cholesteatoma, including those who had undergone previous surgical procedures.
- *Intervention:* Studies in which patients underwent SP with BSC of the EAC with or without simultaneous procedures for bone-anchored hearing aids (BAHA), active middle ear implant (AMEI), middle ear transducer (MET), direct acoustic cochlear stimulator (DACS) or cochlear implant (CI).
- *Comparison and Outcome:* The primary outcomes assessed were healing, failure, complications, and disease recurrence, making a comparison with our case series.
- *Timing:* Studies published up to January 2023 have been included in this literature review.
- *Setting:* Randomized controlled trials (RCT), non-randomized controlled trials (NRCT), prospective or retrospective cohort studies, and case–control studies from community, private, and tertiary care university hospitals were included.

## 3. Results

### 3.1. Study Sample

This retrospective analysis comprised nine patients, including five males and four females. Eight of the patients were diagnosed with chronic cholesteatomatous otitis media, and one presented with atelectatic otitis. The mean age of the patients was 47.3 years, with an age range of 28 to 70 years. All patients in our series reported various degrees of hearing loss. The hearing loss was confirmed by audiometric examination in seven patients. In the two patients with autism spectrum disorder, it was not possible to proceed with the evaluation of the audiometric threshold. Eight patients reported intermittent otorrhea along with persistent or recurrent ear infections. Additionally, four patients suffered from persistent otalgia, and two described symptoms of intermittent vertigo. Three patients had a history of ear surgery. Fibroendoscopic examination of the nose, paranasal sinuses, and nasopharynx revealed no pathological findings. The baseline clinical characteristics of the patients involved in our study are summarized in Table 1.

**Figure 1:**
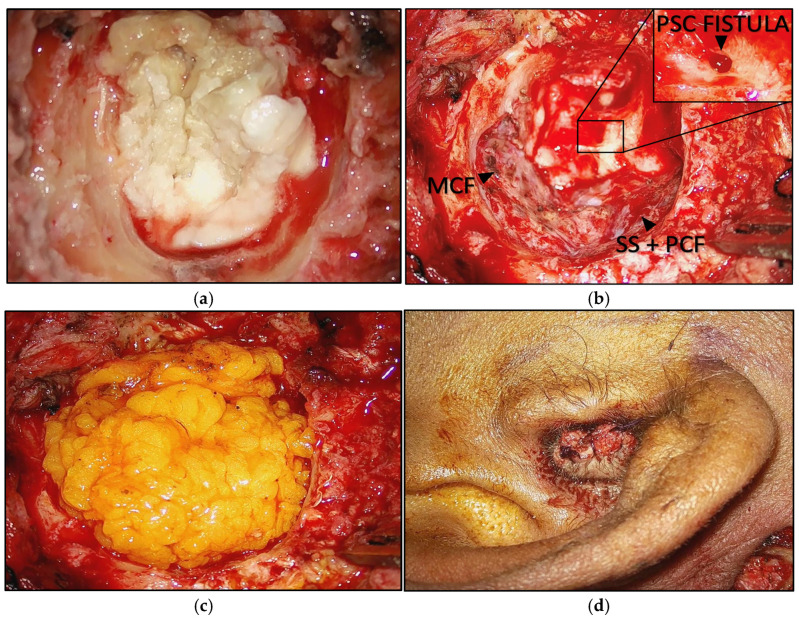
(**a**) Middle ear and mastoid cholesteatoma; (**b**) tympano-mastoid cavity. MCF = middle cranial fossa; SS = sigmoid sinus; PCF = posterior cranial fossa; PSC = posterior semicircular canal. (**c**) Abdominal fat obliteration; (**d**) blind sac closure of the external auditory canal.

In all cases, patients underwent an SP with a BSC of the EAC. Cavity obliteration was performed using abdominal fat grafts and a temporalis muscle flap in all cases. ET obliteration was achieved using cartilage and bone wax for all patients. In addition to these procedures, CI was placed in three patients, and one patient received a BAHA. During the surgeries, conditions such as the absence, erosion, or fixation of the ossicular chain were observed in five patients. Erosion of the FC at various segments was noted in three cases. Additionally, fistulas involving the posterior, anterior, or lateral semicircular canal were detected in two patients. In two instances, there was exposure of the dura mater about the middle or posterior cranial fossa, and one case presented with meningoencephalic herniation. The surgical features of the patients in our study are summarized in Table 2. Figure 1a–d show the intraoperative findings of case number nine.

In our series, none of the patients developed postoperative complications at the surgical or the donor site. In one case (case 9), the patient developed symptoms of vertigo. Treatment with steroids and diazepam was initiated on the first day and led to the complete resolution of symptoms after approximately three days. Patients who received BAHA or CI proceeded with their auditory rehabilitation, which included activation of the processor one month after surgery and subsequent scheduled fittings. These interventions significantly improved auditory performance, as assessed by the speech recognition test and the MATRIX sentence test. Long-term follow-up duration ranged from 2 to 5 years, with no long-term complications reported. MRI conducted one year postoperatively showed no evidence of residual or recurrent disease.

### 3.2. Literature Review

**Figure 2:**
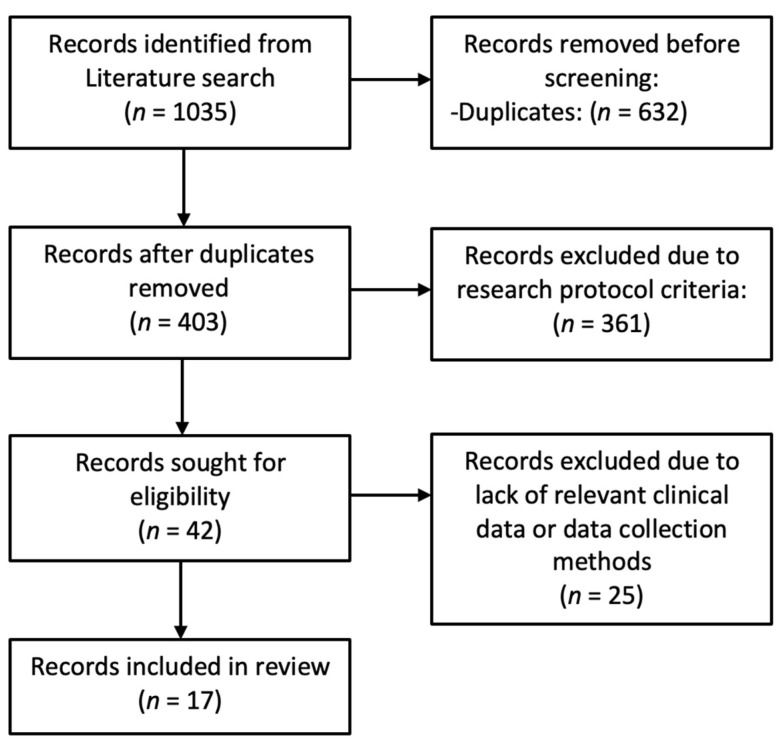
PRISMA 2020 flow diagram.

Our initial literature search yielded 1035 references. By applying the PRISMA 2020 flow diagram, we excluded 632 duplicates, leaving 403 abstracts for review. Subsequently, 361 articles were excluded by the research protocol criteria. The remaining 42 papers were then read in detail by two independent authors. A discussion ensued to reach a consensus on the inclusion or exclusion of each paper for the present study. Ultimately, only 17 papers met the inclusion and exclusion criteria and were incorporated into the review. The most common reasons for exclusion were the initial clinical condition of the treated cases (such as osteoradionecrosis, osteomyelitis, temporal bone fracture, and malignancies of the temporal bone), the types of study design (e.g., case reports), and/or the absence of relevant clinical data. Figure 2 shows the PRISMA 2020 flow diagram. Table 3 and Table 4 show the patients’ clinical characteristics and surgical features from the selected studies.

## 4. Discussion

The most common technique historically used for treating a discharging ear associated with cholesteatoma involved creating a modified radical mastoid cavity through a canal wall–down procedure. However, with the improved understanding of cholesteatoma pathogenesis, this approach is no longer considered the routine option. More conservative techniques are now preferred, as they better address the mechanisms of cholesteatoma development, particularly the role of squamous epithelial migration rather than mucosal metaplasia. This shift also explains the effectiveness of obliteration, in which closure of the middle ear and external auditory canal is the key element, while the obliteration material mainly serves to restore cavity volume and prevent retraction or meningocele. This conceptual evolution is fundamental: recurrence is prevented by removing all squamous epithelium, and residual disease depends on the completeness of petrosectomy.

Ossicular chain reconstruction, which can be performed simultaneously or in a delayed fashion, aims to restore hearing in patients with adequate residual cochlear function. However, patients with an open mastoid cavity require long-term outpatient monitoring and lifestyle modifications to avoid water exposure, which can negatively impact their quality of life. While the incidence of problematic cavities following a canal wall-down procedure is relatively low, about 3% of patients may persistently experience ear discharge despite multiple revision surgeries for disease eradication [24,25]. In 1957, Rambo introduced the trans canal radical mastoidectomy and cavity obliteration using a pedicled temporalis muscle flap. At that time, the rationale of the procedure was to reduce the size of the mastoid cavity and prevent its troublesome consequences. Because the metaplasia theory was still widely accepted, complete elimination of squamous epithelium was not yet understood as the key factor, and otologists were reluctant to perform complete obliteration, and Rambo aimed instead to preserve an air-containing middle ear space connected to the nasopharynx through a patent ET [26]. One year later, the same author reported even higher success with the blind sac closure of the external auditory canal, although total obliteration became fully accepted only after the metaplasia theory was disproved [8]. This historical context is essential to interpret these early studies, as the true pathogenesis of cholesteatoma was not yet recognized.

These findings were later supported by Fritz and Crawford in 1960 and by Bartels and Sheehy in 1981 [8,10]. 1976 Gacek et al. enhanced the technique by incorporating a soft, inferiorly based subcutaneous local flap and an abdominal fat graft [10]. In the early ‘80s, Fisch introduced the concept of SP coupled with ET obliteration used as a complementary step to further isolate the cavity from the nasopharynx. His technique involved the removal of all pneumatization around the otic capsule and the major structures crossing the temporal bone to minimize the risk of epithelial or mucosal remnants and, consequently, the risk of failure [27,28,29,30]. With the subsequent understanding that the metaplasia theory was incorrect, these principles further supported the modern rationale that the success of the procedure depends primarily on the completeness of the petrosectomy and the total removal of squamous epithelium, while ET occlusion remains an accessory step rather than the conceptual foundation of the technique. In 1984, Schuknecht et al. proposed using cartilage, harvested from the tragus or conchal bowl, for ET occlusion [12]. Two years later, Coker et al. suggested using bone wax and cortical bone powder for tubaric isthmus obliteration. This was done after demucosization of the ostium and transposition of fascia and/or muscle. These techniques, however, were based on the erroneous concept of “tubal otorrhea,” which was later abandoned. In the same study, the authors also introduced the practice of exenteration of the mastoid and middle ear in various regions, including the retrosigmoid, retrofacial, posteromedial, posterosuperior, epitympanic, supralabyrinthine, peritubal, and infralabyrinthine areas [4]. Since its inception, the fundamental surgical approach has been standardized and remains largely unchanged. However, there have been variations in the material used to obliterate the tympano-mastoid cavity. In 2016, Lyutenski et al. [21] conducted a comparative analysis of surgical wound dehiscence incidence using various materials to reinforce abdominal fat. This analysis introduced both autologous (periosteal flap, temporalis flap, temporalis fascia) and alloplastic (polydioxanone, allogenic fascia, bovine pericardium) materials and found no significant differences in outcomes [21]. Throughout history, a variety of materials have been used to obliterate the ET, including periosteum, free temporalis muscle graft with fascia, small cartilage fragments from the external auditory canal, bone pate, bioactive ionomeric cement, hydroxyapatite cement, and oxidized regenerated cellulose, with or without supportive materials such as bone wax or fibrin sealant. In 2020, Lyutenski et al. conducted another study in which no substantial differences were observed between autologous or inert alloplastic materials [31].

In our case series, we adopted the classic surgical technique as described by Fisch in the 1980s. We opted for abdominal fat for the obliteration of the tympanomastoid cavity and used cartilage with bone wax for the obliteration of the ET. Several precautions were taken to mitigate the risk of short-term and long-term complications. For the three patients who had undergone previous middle ear surgery, we chose a more posterior skin incision due to the thinning of the retroauricular skin, thereby reducing the risk of fistula formation or wound dehiscence. In eight patients with chronic cholesteatomatous otitis media, we performed a complete removal of the ossicular chain (including the incus, malleus, and stapes superstructure), the annulus, and the tympanic membrane, as well as the complete cellular structure of the tympanomastoid region. This was intended to eliminate any squamous epithelial remnants and prevent recurrent cholesteatoma; this remains a central principle of the current technique and explains why SP with BSC of EAC has achieved high success rates in our series. The stapes superstructure was also removed in order to avoid the onset of postoperative dizziness resulting from the compressive action of the abdominal fat graft. In all patients, obliteration of the ET with cartilage and bone wax followed the demucosization and cauterization of the tubal orifice, enhancing the adhesion of the materials used. Performing BSC without obliteration could simplify the surgical procedure and could reduce operative time and donor-site morbidity. It could also be considered when obliteration materials are unavailable or when minimal surgical manipulation is desired. However, this approach could leave residual air spaces at risk of persistent infection, cholesteatoma recurrence, or cavity-related complications such as retraction pockets. Obliteration of the tympanomastoid cavity and ET, in contrast, could fill potential dead space, reduce the risk of problematic cavities, and facilitate long-term control of residual disease. Therefore, while BSC without obliteration could be considered in selected cases, the combination with cavity and ET obliteration could provide more comprehensive disease eradication and reduce the risk of postoperative complications.

Strict indications and accurate surgical techniques are essential in SP due to the nature of the procedure, which hinders direct visualization of the surgical area during the postoperative period and precludes the possibility of conductive hearing restoration through ossicular chain reconstruction. The primary goal of SP is to eradicate recurrent infection by drilling out multiple air cell tracts and removing large cholesteatomas. This procedure is particularly relevant when these cholesteatomas do not extend deeply into the petrous apex or the internal auditory canal. Between the late 1950s and early 1960s, Rambo’s transcanal radical mastoidectomy with obliteration rapidly gained popularity, particularly in North America, as a treatment for chronic middle ear diseases. However, in a study conducted by Fritz and Crawford in 1960, a high incidence of residual cholesteatoma was observed in patients treated with Rambo’s technique, which often required extensive surgical revision. For this reason, they emphasized that this approach requires greater surgical precision to remove the disease effectively [9]. In 1976, Gacek [10] was the first to provide a comprehensive summary of contemporary indications for total ear obliteration, including chronic middle ear and mastoid diseases in ears where hearing restoration was not possible, as well as in cases with cerebrospinal fluid leaks from meningeal-encephalic herniation in the middle ear. He also advised against total ear obliteration in patients where complete eradication of cholesteatoma or suppurative disease was impossible or in cases where local or systemic conditions may impact wound healing [10]. In 1981, Schuknecht [12] highlighted that obliteration of the middle ear could be performed even when the underlying pathology is chronic cholesteatomatous otitis media, regardless of the patient’s age. Three years later, he expanded the indications to include patients with severe intellectual disability who could not undergo postoperative care without general anesthesia [11,12]. In 1989, Glasscock et al. [24] identified the ideal candidate for total ear obliteration as a patient with a “problematic cavity”. This term refers to an individual who continued to experience otorrhea despite previous mastoidectomy and revisions, typically due to residual cholesteatoma or mucosal disease left from incomplete mastoid cell exenteration. They also proposed that the patient should have impaired or absent cochlear function in the affected ear and normal hearing on the opposite side [24]. Over time, the indications for this procedure have also extended to include other conditions such as petrous bone cholesteatoma (PBC), a rare form of cholesteatoma that affects the petrous portion of the temporal bone and can extend to the clivus, sphenoid sinus, or rhinopharynx, progressively involving vital intracranial structures such as facial nerve, internal carotid artery, sigmoid sinus, jugular bulb, lower cranial nerves, middle and posterior fossa dura, temporal lobe, and cerebellum [32]. In 1995, Gray and Irving [14] recommended SP for patients with chronic middle ear disease who were also candidates for cochlear implantation (CI). They suggested a two-stage strategy: initially performing SP with the BSC of the EAC, followed by CI placement 3 to 6 months later. This sequence was thought to reduce the risks of complications associated with CI by first addressing the chronic infectious [14]. Subsequently, several authors have supported this indication, further establishing that a single-stage strategy with simultaneous SP and CI placement is also feasible when eradicating chronic middle ear disease is confirmed [19,33,34,35,36]. In our series, the 3 patients receiving cochlear implants underwent a single-stage procedure, as complete removal of the underlying chronic condition was achievable. Furthermore, SP has also been recommended when the primary objective is the removal of large tumors without intradural extensions (such as in the case of tympanomastoid paragangliomas, tumors of the facial nerve limited to the tympanic or mastoid segment, and other tumors of the tympanomastoid region). Additionally, it has been utilized for isolating the middle ear and mastoid from the external environment to prevent potential intracranial infection (as seen in cases of temporal bone fractures involving the otic capsule, meningoencephalic herniation, and iatrogenic cerebrospinal fluid leaks). Finally, SP has also been employed to facilitate CI and other implantable device placement in complicated cases involving cochlear obliteration and ossifications, inner ear malformations, and revision cases [6]. In our case series, SP with BSC of the EAC was performed in 16 out of 18 cases (89.9%) to treat chronic cholesteatomatous otitis media. Among these, 3 patients had prior middle ear surgery, 2 were diagnosed with autism, and 2 were candidates for CI placement. The indication for the single case of chronic atelectatic otitis media was the unfavorable anatomy, characterized by cochlear ossification and sigmoid sinus protrusion.

In the analysis of results from the case series within the selected study groups, the most common postoperative complication was surgical wound dehiscence, with a weighted average percentage of 6.5%. This was followed by the occurrence of infections, with a weighted average percentage of 4.9%. Table 5 shows the results observed in the patients included in the selected studies.

The study conducted by Lyutenski et al. found that the development of surgical wound dehiscence is not dependent on using reinforcement materials, such as a temporalis muscle flap, to cover the mastoid area and improve wound closure. Indeed, applying such techniques did not decrease the incidence of dehiscence [21]. In our case series, no patient developed surgical wound dehiscence. We believe that using a more posteriorly placed retroauricular incision, especially in those with previous surgical interventions, may limit such complications. Regarding the incidence of postoperative infections, these are most associated with the healing of the EAC closure site. This association may be because many of these ears had previously undergone meatoplasty, leading to compromised blood supply to the soft tissue of the EAC and, consequently, a higher risk of infection. To maximize the success of the BSC and minimize the risk of postoperative infection, we recommend meticulous care to avoid traction on the skin flap of the EAC and the utilization of a second layer of soft tissue, such as the mastoid periosteal flap, in addition to broad-spectrum antibiotic coverage. As for delayed complications, recurring cholesteatoma is the most frequently observed, with a weighted average percentage of 3%. However, the incidence of this complication has significantly decreased over time. In the past, the surgical procedure was considered risky due to a high recurrence rate of the pathology and the challenge of visualizing the surgical cavity following BSC of the EAC. Moreover, a less aggressive technique that minimized manipulation of the tympano-mastoid cavity was preferred, which increased the likelihood of recurrence. Residual cholesteatoma refers to epithelial remnants that are unintentionally left behind during the primary surgery, while recurrent cholesteatoma develops due to retraction pockets or new epithelial migration over time. This distinction is crucial, as residual cholesteatoma reflects on the completeness of surgical eradication, whereas recurrent disease is more associated with anatomical predisposition or insufficient follow-up. In such specific cases, surgeons prefer a more radical approach that involves removing all cholesteatomatous matrix, squamous epithelium, and pathological mucosa and thoroughly drilling out the tympano-mastoid cells. Although this procedure is generally considered safe today, thanks to advancements such as MRI follow-up with fat-suppression sequences and DWI, the limited size of our case series prevents us from drawing definitive conclusions regarding safety or recurrence rates. In our small cohort, we did not observe any cases of cholesteatoma recurrence, which is consistent with what has been reported in the literature. Nevertheless, our findings should be interpreted with caution, while the broader literature suggests that the procedure appears to be relatively safe.

## 5. Conclusions

Subtotal petrosectomy is a highly beneficial surgical procedure for managing chronic inflammatory conditions of the middle ear. It is particularly effective for treating complex cases where patients do not respond to medical therapy, for individuals who have undergone previous surgical treatments, or for patients with accompanying hearing loss that requires the placement of implantable devices. With precise surgical technique and meticulous postoperative care, it can be stated that the incidence of both immediate and delayed postoperative complications is minimal. However, several limitations of this study should be acknowledged. Firstly, the retrospective design may introduce bias, as data collection relies on historical records. Secondly, the sample size of nine patients is relatively small, limiting the generalizability of the findings. Additionally, the study lacks a control group for comparison, and the absence of a standardized treatment protocol might influence the outcomes. Moreover, the literature review might be influenced by publication bias, as positive results are more likely to be published. Despite these limitations, the study contributes to the existing knowledge on SP with BSC of the EAC. The detailed description of the surgical technique, patient characteristics, and outcomes adds to understanding this procedure’s applicability and success. Further research with larger sample sizes, prospective designs, and long-term follow-ups is warranted to validate the study’s findings and address the identified limitations.

## Figures and Tables

**Table 1:** Study sample clinical characteristics.

Case	Age	Sex	Symptoms	Side	Preoperative Otoscopy	Preoperative PTA	Previous Surgery	Other Features
1	51	M	Hearing loss, otorrhea, persistent infection	Right	Granulation tissue, squamous debris, secretions	NA	-	Autism
2	28	M	Hearing loss, otorrhea, persistent infection	Right	EAC stenosis, granulation tissue, squamous debris, secretions	NA	-	Autism
3	36	M	Hearing loss, otalgia, otorrhea, recurrent infection	Left	Granulation tissue, squamous debris, secretions	Severe CHL	-	-
4	70	F	Hearing loss, otorrhea, recurrent infection	Right	squamous debris, secretions	Severe CHL	Right CWD tympanoplasty	-
5	60	F	Hearing loss	Left	TM atelectasis	Profound SNHL	-	-
6	44	F	Hearing loss, otalgia, otorrhea, recurrent infection	Left	CSF leak, squamous debris	Severe CHL	Left CWD tympanoplasty	-
7	53	F	Hearing loss, otalgia, vertigo, otorrhea, persistent infection	Left	squamous debris, secretions	Anacusis	Left CWU tympanoplasty	EAC stenosis
8	38	M	Hearing loss, otalgia, otorrhea, persistent infection	Right	squamous debris, secretions	Anacusis	-	-
9	46	M	Hearing loss, vertigo, otorrhea, persistent infection	Right	Granulation tissue, squamous debris, secretions	Severe CHL	-	-

PTA = pure-tone audiometry, M = male, F = female, NA = not available, EAC = external auditory canal, CHL = conductive hearing loss, CWU = canal-wall up, CWD = canal-wall down, TM = tympanic membrane, SNHL = sensori-neural hearing loss.

**Table 2:** Study sample intraoperative surgical features.

Case	Intraoperative Features	Intervention	Cavity Obliteration	ET Obliteration
1	-	SP + BSC	Abdominal fat	Cartilage + bone wax
2	Eroded incus	SP + BSC	Abdominal fat	Cartilage + bone wax
3	No stapes, no incus, eroded FC (tympanic tract)	SP + BSC	Abdominal fat	Cartilage + bone wax
4	-	SP + BSC + BAHA	Abdominal fat	Cartilage + bone wax
5	No stapes, no incus	SP + BSC + CI	Abdominal fat	Cartilage + bone wax
6	MCF dura exposed, Meningo-encephalic hernia	SP + BSC	Abdominal fat	Cartilage + bone wax
7	No stapes, fixed and eroded incus, eroded FC (tympanic tract), ASC and LSC fistulas	SP + BSC + CI	Abdominal fat	Cartilage + bone wax
8	-	SP + BSC + CI	Abdominal fat	Cartilage + bone wax
9	Eroded stapes, no incus, eroded FC (2nd genu, tympanic tract, mastoid tract), exposed MCF and PCF dura, exposed SS, PSC, and LSC fistulas	SP + BSC	Abdominal fat	Cartilage + bone wax

ET = eustachian tube, SP = subtotal petrosectomy, BSC = blind sac closure, FC = fallopian canal, BAHA = bone-anchored hearing aids, CI = cochlear implant, MCF = middle cranial fossa, PCF = posterior cranial fossa, ASC = anterior semicircular canal, LSC = lateral semicircular canal, PSC = posterior semicircular canal.

**Table 3:** Clinical characteristics of the patients included in the selected studies.

Author	Cases (*n* of Ears)	Mean Age	Previous Surgery	Indications	Symptoms	Other Features
Rambo, J.H.T., 1958 [8]	4 (4)	NA	NA	COM cholesteatoma (NA)	NA	Severe- profound SNHL (3)
Fritz, M.H. et al., 1960 [9]	157 (157)	NA	NA	COM cholesteatoma (NA)	NA	NA
Gacek, R.R. et al., 1976 [10]	6 (6)	42	6	COM cholesteatoma (3)	recurrent infections (5) CSF leak (2) otorrhea (1)	Severe- profound SNHL (5)
Bartels, L.J. et al., 1981 [11]	24 (24)	NA	23	COM cholesteatoma (8) MET (2)	recurrent infections (16) otorrhea (6) otalgia (3) facial palsy (3)	Severe- profound SNHL (7)
Schuknecht, H.F. et al., 1984 [12]	44 (44)	40.5	30	COM cholesteatoma (27) meningitis (1)	otorrhea (39) otalgia (11) facial palsy (9) vertigo (6)	EAC stenosis (11) Severe- profound SNHL (39)
Parikh, A.A. et al., 1994 [13]	10 (10)	43.8	10	COM cholesteatoma (6)	recurrent infection (10) otorrhea (10) otalgia (2) facial palsy (1)	Severe- profound SNHL (7)
Gray, F. et al., 1995 [14]	4 (4)	NA	4	COM cholesteatoma (2)	recurrent infection (2) otorrhea (2)	Severe- profound SNHL (4)
Kos, M.I. et al., 2006 [15]	46 (46)	NA	32	COM cholesteatoma (32)	vertigo (11) facial palsy (3)	Severe- profound SNHL (46)
Sanna, M. et al., 2008 [6]	53 (53)	57	44	COM cholesteatoma (43)	otorrhea (1) CSF leak (17)	EAC stenosis (8) Severe- profound SNHL (24)
Verahert, N. et al., 2013 [16]	22 (22)	57	22	COM cholesteatoma (15)	otorrhea (8)	Severe- profound SNHL (22)
Patel, M. et al., 2014 [17]	29 (32)	54	NA	COM cholesteatoma (21)	otorrhea (11)	Severe- profound SNHL (24)
Magliulo, G. et al., 2015 [18]	10 (10)	47.8	4	COM cholesteatoma (1)	recurrent infections (9) otorrhea (6)	NA
Szymanski et al., 2016 [19]	19 (19)	54	7	COM cholesteatoma (3)	otorrhea (6)	Severe- profound SNHL (19)
Schwab et al., 2016 [20]	4 (4)	NA	4	COM cholesteatoma (0)	otorrhea (4) recurrent infections (4)	NA
Lyutenski et al., 2016 [21]	199 (212)	65	212	COM cholesteatoma (NA)	NA	Severe- profound SNHL (101)
Kraus et al., 2020 [22]	12 (12)	NA	11	COM cholesteatoma (2)	otorrhea (12)	NA
Lee et al., 2020 [23]	25 (25)	63.8	NA	COM cholesteatoma (7)	otorrhea (13)	Severe- profound SNHL (25)

NA = not available, COM = chronic otitis media, SNHL = sensori-neural hearing loss, CSF = cerebrospinal fluid, MET = middle ear tuberculosis, EAC = external auditory canal.

**Table 4:** Surgical features of the patients included in the selected studies.

Author	Surgical Procedure	Intraoperative Features	Cavity Obliteration	ET Obliteration
Rambo, J.H.T., 1958 [8]	SP + BSC (4)	NA	temporalis muscle flap	no
Fritz, M.H. et al., 1960 [9]	SP + BSC (157)	labyrinth fistula (2)	temporalis muscle flap	no
Gacek, R.R. et al., 1976 [10]	SP + BSC (6)	meningo-encephalic herniation (2)	abdominal fat + soft tissue	no
Bartels, L.J. et al., 1981 [11]	SP + BSC (24)	NA	abdominal fat, temporalis muscle fascia, bone paté, Palva flap	no
Schuknecht, H.F. et al., 1984 [12]	SP + BSC (44)	abscess (1) meningo-encephalic herniation (3) labyrinth fistula (9) LSC dehiscence (17)	abdominal fat, free muscle, muscle flap, myocutaneous flap	cartilage
Parikh, A.A. et al., 1994 [13]	SP + BSC (10)	labyrinth fistula (3)	abdominal fat	bone paté, bone wax, oxidized regenerated cellulose
Gray, F. et al., 1995 [14]	SP + BSC + CI (4)	NA	abdominal fat + temporalis muscle fascia	NA
Kos, M.I. et al., 2006 [15]	SP + BSC (46)	NA	abdominal fat	cartilage
Sanna, M. et al., 2008 [6]	SP + BSC (53)	exposed dura mater (36) meningo-encephalic herniation (30) eroded FC (18) labyrinth fistula (14)	abdominal fat	bone wax
Verahert, N. et al., 2013 [16]	SP + BSC (4) SP + BSC + CI (2) SP + BSC + AMEI (16)	NA	abdominal fat + periosteal flap	bone wax + oxidized regenerated cellulose
Patel, M. et al., 2014 [17]	SP + BSC (24) SP + BSC + CI (6) SP + BSC + BAHA (2)	NA	temporalis muscle flap + temporalis muscle fascia + periosteal flap	temporal muscle
Magliulo, G. et al., 2015 [18]	SP + BSC + BAHA (10)	meningo-encephalic herniation (1)	abdominal fat + periosteal flap	bone wax + temporal muscle
Szymanski et al., 2016 [19]	SP + BSC + CI (19)	eroded FC (1)	abdominal fat + temporalis muscle flap	bone wax
Schwab et al., 2016 [20]	SP + BSC + DACS (4)	NA	abdominal flap	NA
Lyutenski et al., 2016 [21]	SP + BSC (43) SP + BSC + CI (101) SP + BSC + AMEI (57) SP + BSC + MET (7) SP + BSC + DACS (4)	NA	abdominal fat + periosteal flap, abdominal fat + periosteal flap + temporalis muscle fascia + temporalis muscle flap, abdominal fat + polydioxanone/allogenic fascia/bovine pericardium	bone wax + oxidized regenerated cellulose
Kraus et al., 2020 [22]	SP + BSC (12)	NA	abdominal fat, fibrin glue	periosteum, cartilage
Lee et al., 2020 [23]	SP + BSC + CI (25)	NA	abdominal fat, temporalis muscle flap	bone wax, soft tissue, fibrin glue

SP = subtotal petrosectomy, BSC = blind sac closure, NA = not available, LSC = lateral semicircular canal, CI = cochlear implant, FC = fallopian canal, AMEI = active middle ear implant, BAHA = bone-anchored hearing aids, MET = middle ear transducer, DACS = direct acoustic cochlear stimulator.

**Table 5:** Results observed in the patients included in the selected studies.

Author	Postoperative Complications % (*n*)	Delayed Complications % (*n*)	Failure % (*n*)	Reinterventions % (*n*)
Rambo, J.H.T., 1958 [8]	0% (0)	0% (0)	0% (0)	NA
Fritz, M.H. et al., 1960 [9]	0% (0)	recurrent cholesteatoma 3.8% (6)	0% (0)	NA
Gacek, R.R. et al., 1976 [10]	0% (0)	0% (0)	0% (0)	0% (0)
Bartels, L.J. et al., 1981 [11]	infection 12.5% (3) SNHL 8.3% (2)	recurrent infection 8.3% (2) recurrent cholesteatoma 4.2% (1) delayed facial palsy 4.2% (1)	4.2% (1)	16.7% (4)
Schuknecht, H.F. et al., 1984 [12]	infection 20.4% (9) vertigo 9% (4) seroma 6.8% (3) hematoma of donor site 4.5% (2) incisional granulation 2.3% (1) transient facial palsy 2.3% (1)	recurrent cholesteatoma 6.8% (3) delayed facial palsy 2.3% (1)	NA	11.4% (5)
Parikh, A.A. et al., 1994 [13]	infection 30% (3) vertigo 20% (2)	delayed facial palsy 20% (2)	0% (0)	30% (3)
Gray, F. et al., 1995 [14]	0% (0)	recurrent cholesteatoma 25% (1)	0% (0)	NA
Kos, M.I. et al., 2006 [15]	infection 17.4% (8)	recurrent cholesteatoma 8.7% (4) recurrent infection 2.2% (1)	1 (2.2%)	23.9% (11)
Sanna, M. et al., 2008 [6]	SNHL 9.4% (5) post-aural fistula 1.9% (1)	recurrent cholesteatoma 1.9% (1) residual cholesteatoma 1.9% (1)	0% (0)	3.8% (2)
Verahert, N. et al., 2013 [16]	wound dehiscence 18.2% (4) transient facial palsy 4.5% (1) hematoma of the donor site 4.5% (1)	0% (0)	13.6% (3)	13.6% (3)
Patel, M. et al., 2014 [17]	infection 25% (8) incisional granulation 3.1% (1) wound dehiscence 3.1% (1) seroma 3.1% (1)	recurrent cholesteatoma 15.6% (5) residual cholesteatoma 15.6% (5)	0% (0)	15.6% (5)
Magliulo, G. et al., 2015 [18]	0% (0)	0% (0)	0% (0)	0% (0)
Szymanski et al., 2016 [19]	0% (0)	0% (0)	0% (0)	0% (0)
Schwab et al., 2016 [20]	0% (0)	0% (0)	0% (0)	0% (0)
Lyutenski et al., 2016 [21]	infection 1.4% (3) wound dehiscence 16% (34)	entrapped cholesteatoma 2.4% (5) CI extrusion 0.5% (1)	0% (0)	16% (34)
Kraus et al., 2020 [22]	wound dehiscence 40% (3)	0% (0)	0% (0)	40% (3)
Lee et al., 2020 [23]	wound dehiscence 4% (1)	CI extrusion 8% (2)	0% (0)	12% (3)
Our series	vertigo 11.1% (1)	0% (0)	0% (0)	0% (0)

NA = not available, SNHL = sensori-neural hearing loss.

## Data Availability

Restrictions apply to the availability of these data, which are used under license for the current study and so are not publicly available.

## Abbreviations

The following abbreviations are used in this manuscript:

PRISMA	Preferred Reporting Items for Systematic Reviews and Meta-analyses
PICOTS	Population, Intervention, Comparison, Outcome, Timing, and Setting
SP	Subtotal Petrosectomy
ET	Eustachian Tube
BSC	Blind Sac Closure
EAC	External Auditory Canal
CT	Computed Tomography
MRI	Magnetic Resonance Imaging
DWI	Diffusion-Weighted Imaging
SEMAC	Slice Encoded Metal Artifact Correction
MAVRIC	Multi-Acquisition Variable-Resonance Image Combination
CWD	Canal-Wall Down
FC	Fallopian Canal
CI	Cochlear Implant
BAHA	Bone-Anchored Hearing Aids
AMEI	Active Middle Ear Implants
MET	Middle Ear Transducer
DACS	Direct Acoustic Cochlear Stimulator
RCT	Randomized Controlled Trial
NRCT	Non-Randomized Controlled Trial

## References

[1] Prasad S.C., Roustan V., Piras G., Caruso A., Lauda L., Sanna M. (2017). Subtotal petrosectomy: Surgical technique, indications, outcomes, and comprehensive review of literature. Laryngoscope.

[2] Immordino A., Sireci F., Lorusso F., Martines F., Dispenza F. (2022). The Role of Cartilage-perichondrium Tympanoplasty in the Treatment of Tympanic Membrane Retractions: Systematic Review of the Literature. Int. Arch. Otorhinolaryngol..

[3] Rambo J.H. (1965). Musculoplasty: Advantages and disadvantages. Ann. Otol. Rhinol. Laryngol..

[4] Coker N.J., Jenkins H.A., Fisch U. (1986). Obliteration of the middle ear and mastoid cleft in subtotal petrosectomy: Indications, technique, and results. Ann. Otol. Rhinol. Laryngol..

[5] Cazzador D., Franz L., Tealdo G., Carobbio A.L.C., Ferraro M., Mazzoni A., Marioni G., Zanoletti E. (2023). Survival Outcomes in Squamous Cell Carcinoma of the External Auditory Canal: A Systematic Review and Meta-Analysis. J. Clin. Med..

[6] Sanna M., Dispenza F., Flanagan S., De Stefano A., Falcioni M. (2008). Management of chronic otitis by middle ear obliteration with blind sac closure of the external auditory canal. Otol. Neurotol..

[7] Page M.J., McKenzie J.E., Bossuyt P.M., Boutron I., Hoffmann T.C., Mulrow C.D., Shamseer L., Tetzlaff J.M., Akl E.A., Brennan S.E. (2021). The PRISMA 2020 statement: An updated guideline for reporting systematic reviews. BMJ.

[8] Rambo J.H. (1958). Primary closure of the radical mastoidectomy wound: A technique to eliminate postoperative care. Laryngoscope.

[9] Fritz M.H., Crawford G.B. (1960). An evaluation of the Rambo primary closure of the radical mastoidectomy wound. Trans. Am. Acad. Ophthalmol. Otolaryngol..

[10] Gacek R.R. (1976). Mastoid and middle ear cavity obliteration for control of otitis media. Ann. Otol. Rhinol. Laryngol..

[11] Bartels L.J., Sheehy J.L. (1981). Total obliteration of the mastoid, middle ear, and external auditory canal. A review of 27 cases. Laryngoscope.

[12] Schuknecht H.F., Chandler J.R. (1984). Surgical obliteration of the tympanomastoid compartment and external auditory canal. Ann. Otol. Rhinol. Laryngol..

[13] Parikh A.A., Brookes G.B. (1994). Subtotal petrosectomy with external canal overclosure in the management of chronic suppurative otitis media. J. Laryngol. Otol..

[14] Gray R.F., Irving R.M. (1995). Cochlear implants in chronic suppurative otitis media. Am. J. Otol..

[15] Kos M.I., Chavaillaz O., Guyot J.P. (2006). Obliteration of the tympanomastoid cavity: Long term results of the Rambo operation. J. Laryngol. Otol..

[16] Verhaert N., Mojallal H., Schwab B. (2013). Indications and outcome of subtotal petrosectomy for active middle ear implants. Eur. Arch. Otorhinolaryngol..

[17] Patel M., Loan F.L., Lyon J.R., Bird P.A. (2014). Blind sac closure of the external auditory canal for chronic middle ear disease. Otol. Neurotol..

[18] Magliulo G., Turchetta R., Iannella G., Valperga di Masino R., de Vincentiis M. (2015). Sophono Alpha System and subtotal petrosectomy with external auditory canal blind sac closure. Eur. Arch. Otorhinolaryngol..

[19] Szymański M., Ataide A., Linder T. (2016). The use of subtotal petrosectomy in cochlear implant candidates with chronic otitis media. Eur. Arch. Otorhinolaryngol..

[20] Schwab B., Kludt E., Maier H., Lenarz T., Teschner M. (2016). Subtotal petrosectomy and Codacs™: New possibilities in ears with chronic infection. Eur. Arch. Otorhinolaryngol..

[21] Lyutenski S., Schwab B., Lenarz T., Salcher R., Majdani O. (2016). Impact of the surgical wound closure technique on the revision surgery rate after subtotal petrosectomy. Eur. Arch. Otorhinolaryngol..

[22] Kraus M., Hassannia F., Bergin J.M., Al Zaabi K., Rutka J.A. (2020). Long-Term Outcomes from Blind Sac Closure of the External Auditory Canal: Our Institutional Experience in Different Pathologies. J. Int. Adv. Otol..

[23] Lee S., Lee J.B., Chung J.H., Park K.W., Choi J.W. (2020). Surgical outcomes of simultaneous cochlear implantation with subtotal petrosectomy. Auris Nasus Larynx.

[24] Glasscock M.E., Poe D.S., Johnson G.D., Ragheb S., Tos M., Thomsen J., Peitersen E. (1989). Surgical management of the previously operated chronic ear. Cholesteatoma and Mastoid Surgery.

[25] Immordino A., Salvago P., Sireci F., Lorusso F., Immordino P., Saguto D., Martines F., Gallina S., Dispenza F. (2023). Mastoidectomy in surgical procedures to treat retraction pockets: A single-center experience and review of the literature. Eur. Arch. Otorhinolaryngol..

[26] Rambo J.H. (1957). A new operation to restore hearing in conductive deafness of chronic suppurative origin. AMA Arch. Otolaryngol..

[27] Fisch U. (1982). Infratemporal fossa approach for glomus tumors of the temporal bone. Ann. Otol. Rhinol. Laryngol..

[28] Fisch U., Pillsbury H.C. (1979). Infratemporal fossa approach to lesions in the temporal bone and base of skull. Arch. Otolaryngol..

[29] Fisch U. (1983). The infratemporal fossa approach for nasopharyngeal tumors. Laryngoscope.

[30] Fisch U., Oldring D.J., Senning A. (1980). Surgical therapy of internal carotid artery lesions of the skull base and temporal bone. Otolaryngol. Head Neck Surg..

[31] Lyutenski S., El-Saied S., Schwab B. (2020). Impact of occlusive material and cochlea-carotid artery relation on eustachian tube occlusion in subtotal petrosectomy. Laryngoscope Investig. Otolaryngol..

[32] Omran A., De Donato G., Piccirillo E., Leone O., Sanna M. (2006). Petrous bone cholesteatoma: Management and outcomes. Laryngoscope.

[33] Henseler M.A., Polanski J.F., Schlegel C., Linder T. (2014). Active middle ear implants in patients undergoing subtotal petrosectomy: Long-term follow-up. Otol. Neurotol..

[34] Sanna M., Free R., Merkus P., Falcioni M. (2016). Subtotal Petrosectomy in Cochlear Implantation Surgery for Cochlear and Other Auditory Implants.

[35] Immordino A., Lorusso F., Sireci F., Dispenza F. (2023). Acute pneumolabyrinth: A rare complication after cochlear implantation in a patient with obstructive sleep apnoea on CPAP therapy. BMJ Case Rep..

[36] Dispenza F., Immordino A., De Stefano A., Sireci F., Lorusso F., Salvago P., Martines F., Gallina S. (2022). The prognostic value of subjective nasal symptoms and SNOT-22 score in middle ear surgery. Am. J. Otolaryngol..

